# Improving Cardiovascular Risk in Postmenopausal Women with an (−)-Epicatechin-Based Nutraceutical: A Randomly Assigned, Double-Blind vs. Placebo, Proof-of-Concept Trial

**DOI:** 10.3390/jcm13010195

**Published:** 2023-12-29

**Authors:** Nayelli Nájera, Miguel Ortíz-Flores, Javier Pérez-Durán, Enrique Reyes-Muñoz, José Romo-Yañez, Guillermo Ortiz-Luna, Francisco Villarreal, Eduardo Meaney, Guillermo Ceballos, Araceli Montoya-Estrada

**Affiliations:** 1 Sección de Estudios de Posgrado, Escuela Superior de Medicina, Instituto Politécnico Nacional, Mexico City 11340, Mexico; nnajerag@ipn.mx (N.N.); angelito_coa@hotmail.com (M.O.-F.);; 2 Reproductive and Perinatal Health Research Department, National Institute of Perinatology, Ministry of Health, Mexico City 11000, Mexico; 3 Coordination of Gynecological and Perinatal Endocrinology, National Institute of Perinatology, Ministry of Health, Mexico City 11000, Mexico; dr.enriquereyes@gmail.com (E.R.-M.);; 4 Peri and Postmenopause Clinic, National Institute of Perinatology, Ministry of Health, Mexico City 11000, Mexico; 5 School of Medicine, University of California San Diego, San Diego, CA 92093, USA; fvillarr@health.ucsd.edu

**Keywords:** cardiovascular risk, lipoprotein subfractionation profile, epicatechin, (−)-epicatechin-enriched cacao

## Abstract

Background: Age-adjusted rates of cardiovascular disease (CVD) are higher in men than in women. CVD risk-factor outcomes are underrecognized, underestimated, and undertreated in women because the clinical expressions in women differ from those of men. There are no universally accepted recommendations on what to do in women when the values of fasting glucose, blood pressure, and lipids are only slightly altered or at borderline values. We reported the positive effects on CVD risk markers using cacao by-products, showing that alternative approaches can be used to prevent cardiovascular disease in women. The objective was to evaluate the changes in lipoprotein subfractions induced by three months of treatment with an epicatechin-enriched cacao supplement. Methods: A double-blind, placebo-controlled proof-of-concept study was developed to evaluate the effects of 3 months of treatment with an (−)-epicatechin-enriched cacao supplement on lipoprotein subfractions. Results: The usual screening workshop for postmenopausal women could be insufficient and misleading. Assessing the effect of a (−)-epicatechin-enriched cacao supplement employing a lipoprotein subfractionation profile analysis suggests a decrease in cardiovascular risk. Conclusions: A simple, low-cost, safe (−)-epicatechin-enriched cacao supplement product can improve the cardiovascular risk in postmenopausal women.

## 1. Introduction

Cardiovascular disease (CVD) is the leading cause of mortality in both genders [1]. In 2022, there were 200,535 defunctions due to heart disease registered in Mexico, with a national rate of 155.9 per 100,000 inhabitants; 76.4% of these deaths were due to ischemic heart disease [2]. Although age-adjusted rates of CVD are higher in men than in women, in the latter, these diseases are also of fundamental importance as a source of both death and disability [3]. Unfortunately, cardiovascular (CV) risk factors and ischemic heart disease (IHD) outcomes are often underrecognized, underestimated, and undertreated in women, due to deeply rooted prejudices and because the clinical expressions of IHD in women differ from those of men [4,5,6].

The National Survey of Health and Nutrition (ENSANUT 2022) reported a prevalence of 75.2% of overweight or obese individuals in the population over 20 years. This trait was more marked in women (76.8%) than men (73.5%). Abdominal adiposity was also more prominent in women (87.9%) than in men (73.9%). While obesity predominated in women (41% vs. 32.3%), being overweight was more frequent in men (41.2% vs. 35.8%) [7]. Obesity/overweight (O/O), mainly in adults, increases the risk of developing diabetes, high blood pressure, dyslipidemia, ischemic heart disease, stroke, chronic kidney disease, and many other dangerous comorbid conditions [8,9,10]. O/O women with the so-called metabolic syndrome (MS) are known to be at high risk for CVD, still more significantly than their male counterparts [11]. The prevalence of MS increases in the menopause period [12], which partially explains the acceleration and greater severity of CVD in that stage of a woman’s life [13]. As abdominal obesity, often accompanied by binomial insulin resistance/hyperinsulinism, is the more frequent cause of MS, many climacteric women have this clinical complex. A progressive aggravation of MS manifestations has been observed from pre- to trans- and postmenopause stages [14]. Hormone replacement therapy is not currently accepted in the primary and secondary prevention of atherosclerotic cardiovascular disease (ASCVD) [15]. Although it can have some benefits in some features of the syndrome in menopausal and postmenopausal women [16], there is no solid evidence that its benefits outweigh its risks [17]. The current guidelines advise that CV risk has to be screened in menopausal and postmenopausal women and that all CV risk factors must be reduced with the proper treatment, including pharmacotherapy if indicated [15,17]. To our knowledge, there are no universally accepted recommendations on what to do in these women when the values of fasting glucose, blood pressure, and lipids are only slightly altered or at borderline values. When this situation is associated with O/O, the only recommendation is weight loss, which entails various known and challenging obstacles [18].

On the other hand, we have reported positive effects on cardiometabolic risk markers using (−)-epicatechin on postprandial fat and carbohydrate metabolism in normal and overweight subjects; in that study, we explored the respiratory quotient (RQ) to determine the primary source of energy metabolism. We showed that (−)-epicatechin lowers the RQ ratio, reflecting increased fat oxidation [19]; we also showed that the triglyceride/HDLc ratio and cardiometabolic profile of subjects with hypertriglyceridemia improves [20]. Also, using cacao by-products, we showed a decrease in hypertriglyceridemia and the TG/HDL index in overweight women, suggesting that alternative approaches can be used as tools in cardiovascular disease prevention in women [21].

The present work has two main objectives: (1) to compare the CV risks in postmenopausal women obtained through traditional biochemical tests, measuring risk through several scales and with lipoprotein fractionation (the ion mobility), and (2) to evaluate the changes in lipoprotein subfractions induced by three months of treatment with an epicatechin-enriched cacao supplement.

## 2. Materials and Methods

**Figure 1:**
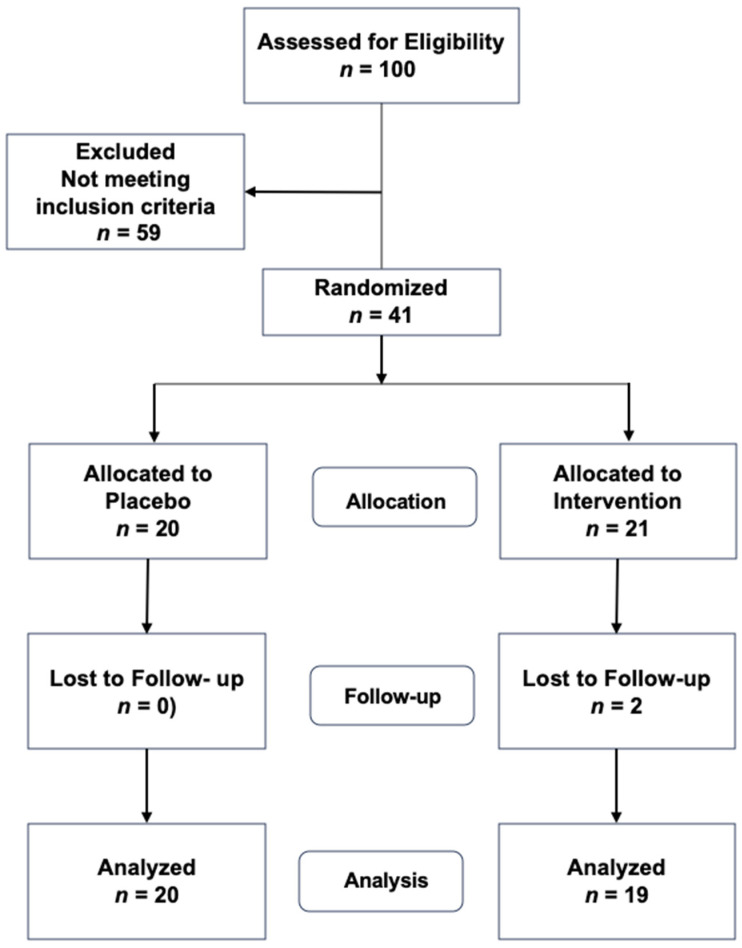
Flow chart.

Study design: The study protocol was approved by the Internal Review Board of INPer (ID: 2020-1-29), and all subjects signed an approved consent to participate. This was a single-center, double-blind, placebo-controlled proof-of-concept study evaluating the effects of 3 months of treatment with an (−)-epicatechin-enriched cacao supplement on lipoprotein subfractions (Figure 1). Considering a triglyceride decrement of 30 ± 2% in treated and 5% in placebo groups with a power of 0.85, a sample size of 18 subjects per group was calculated.

The protocol was registered as NCT05158673 (www.clinicaltrials.gov).

Inclusion criteria: Women aged 50–60 years old with a diagnosis of MS, with at least 3 of the following parameters: abdominal circumference > 80 cm; triglycerides ≥ 150 mg/dL; HDL-c < 50 mg/dL; glucose ≥ 100 mg/dL; blood pressure ≥ 130/85 mm Hg.

Exclusion criteria: Chronic diseases, use of hormone replacement therapy, statins, antihypertensives, antihyperglycemic agents, multivitamin supplements, tobacco or alcohol consumption three months before the inclusion.

Initial screening and treatment protocol: A convenience sample of participants was assembled as it is a proof-of-concept study. Of the 100 women who attended the invitation to participate, forty-one women fulfilled the inclusion criteria. They were randomly allocated into a placebo (*n* = 20) or an active intervention (*n* = 21) group. Women and healthcare providers were blinded to treatments. Patients received a flask marked A or B, containing 60 capsules of placebo or 500 mg of a mixture of cacao flour and 15 mg of free (−)-epicatechin. Patients were instructed to ingest one pill twice daily before meals and assist after 30 days to receive a new flask. Treatments were provided for three months.

Anthropometric measures: Body weight was measured with a SECA balance, and height was measured using a stadimeter. Body mass index (BMI) was calculated as kg/m^2^, and abdominal circumference in centimeters was obtained using an anthropometric tape. A blood pressure (BP) measurement was obtained using an electronic sphygmomanometer, following standard recommendations [22].

Serum biochemical analysis: The evaluations were performed at the beginning and the end of the trial. The follow metabolic parameters were glucose (mg/dL), total cholesterol (mg/dL), high-density cholesterol (HDL-c, mg/dL), and triglycerides (TG, mg/dL) evaluated by the institutional central laboratory using traditional methodologies. LDL was calculated using the Friedewald formula considering TG less than 300 mg/dL [23]. Malondialdehyde (MDA), a marker of lipid oxidation, was measured using 1-methyl-2-phenylindole. This assay is based on the reaction of 1-methyl-2-phenylindole (MPI) (Sigma-Aldrich) with MDA in the presence of 4-hydroxyalkenals, with acidic conditions, to produce a blue/purple chromophore that was evaluated spectrophotometrically by measuring the absorbance at 586 nm [24].

Analysis of lipoprotein fractionation: Lipoprotein particles and separation based on size and count were analyzed using gas-phase electrophoresis [25] in a private laboratory (Quest Laboratories). Certifications available at https://www.questdiagnostics.com/our-company/about-us/licenses-accreditations, accessed on 24 December 2023

10-year cardiovascular risk: Absolute CV risk was estimated using, in the first place, the ASCVD risk estimator plus from the American College of Cardiology [26,27]. The system considers age, gender, ethnicity, total cholesterol (TC), cholesterol of low-density lipoproteins (LDL-c), the cholesterol of high-density lipoproteins (HDL-c), triglycerides (TGs), systolic blood pressure (SBP), diastolic blood pressure (DBP), tobacco consumption, hypertension, diabetes, and the use of statins or aspirin. With the regression equations of pooled data of cohorts comprehending white and black persons of both genders, the scale considers low risk (<5%), borderline risk (5% to 7.4%), intermediate risk (7.5% to 19.9%), and high risk (≥20%). CV risk was also screened using the TG/HDL index [28] and the Lindavista score (LS, derived from the Lindavista primary prevention program data) on a middle-class mestizo Mexican population sample [29]. Because there are no universally applicable TG/HDL index cut-off values, as it is modified by gender, ethnicity, comorbidities, and other factors, every population must establish the proper applicable risk categories. In previous work, we have established that in a sample of middle-class mestizo Mexicans, the cut-off values of <3.3, 3.4–4.6, 4.7–6, and >6 corresponded to low, borderline, intermediate, and high-risk coinciding with the LS scores of <4.9, 5–8.9, 9–12.9, and >13 [30].

Endothelial dysfunction markers: TNF-α [31] and syndecan-1 [32], indirect glycocalyx/endothelial dysfunction markers, were measured using ELISA Kits (TNF-α, BMS223-4, Thermo Fisher Scientific, Waltham, MA, USA; Syndecan-1, ab46506, Abcam, UK). Polysulfurs (as markers of the endothelial hyperpolarization relaxing factor [33]) were measured using the assay conditions described by Ikeda M [34]. The spectrophotometric assay was read at 670 nm.

### Statistical Methods

Results are represented as the mean ± standard error of the mean. Statistical analysis was conducted using GraphPad Prism version 10.00 for Mac (GraphPad Software Inc., San Diego, CA, USA). A paired t-test was used to compare treatment-induced changes. In the case of the comparison of two groups, a t-student test was used. A value of *p* < 0.05 was considered statistically significant.

## 3. Results

Table 1 displays the data concerning the relationship between CV risk and the size and number of lipoprotein particles [35].

Table 2 shows the biochemical variable characteristics at the beginning of the trial (inclusion).

Table 3 shows the differences in the placebo and experimental groups at the study’s base and end. It was observed in both groups a marginal decrease in body weight (1.3 and 1.05 kg, respectively), BMI (0.6 and 1.23 units each), and waist circumference (1.65 and 1.74 cm in each one), but all were statistically significant. In contrast, there were no observed substantial changes in all other variables except in the concentration of MDA, which diminished an important (but nonsignificant) 39% in the experimental group, and in the value of LDL-c, which increased in both groups but only significantly in the placebo group. The modifications of other lipids, glycemia, and blood pressure were insubstantial.

**Figure 2:**
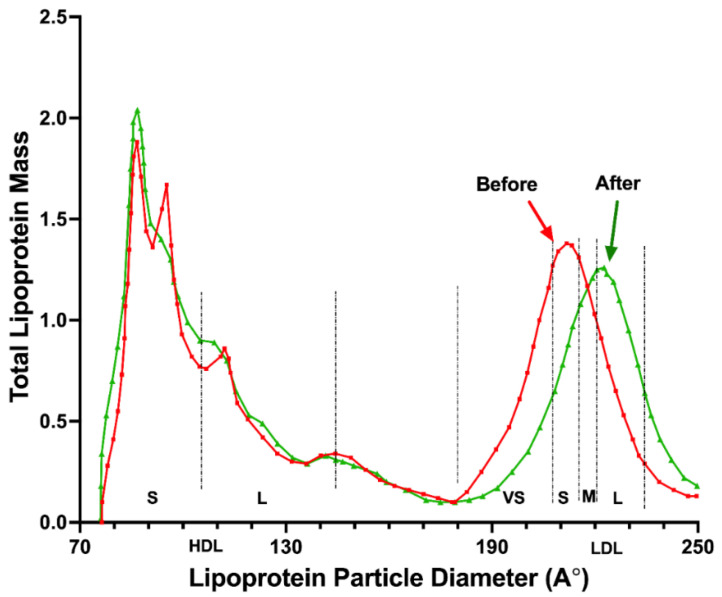
Representative image of the displacement of the size peak of the mass of LDL to the right, indicating that the epicatechin-enriched treatment recomposed the proportion of the LDL subclasses, decreasing the small and more atherogenic ones and increasing the large and buoyant with less atherogenic power.

In Table 4, the data from the lipoprotein fractional analysis are shown. Although there were no remarkable differences in the concentrations of all lipids and lipoproteins in both study groups, the fractionation study revealed a significant reduction in the LDL particles of small and very small size in the experimental group. In contrast, the number of medium and large particles increased. On the contrary, large LDL particles decreased in the placebo group. Moreover, the LDL pattern in the intervention group was displaced to the right, comparing the basal data against the final data (Figure 2 shows a representative pattern change), meaning a positive increase in large LDL with a decrease in very small and small LDL associated with the progression of atherosclerotic processes.

Table 4 also shows that an increment in the number and size of the HDL particles occurred in the experimental group.

**Figure 3:**
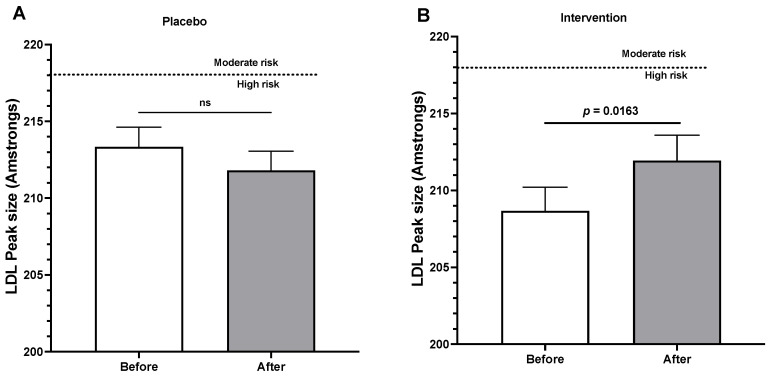
(**A**) LDL peak sizes before and after three months of placebo treatment. (**B**) LDL peak sizes before and after three months of epicatechin-enriched treatment. Data are expressed as the mean ± SEM, and a paired *t*-test was performed in each case. ns: non-statistically significant differences.

Figure 3A shows that the placebo induces no significant changes in LDL peak size; however, intervention (Figure 3B) induced a significant increase in LDL peak size, suggesting a change toward a decrease in cardiovascular risk.

In Table 5, the risk assessment data are displayed. The ACC/AHA scores of the two groups were small, signaling a low CV risk. In contrast, both groups’ mean TG/HDL index values corresponded to the second and third risk categories from the Lindavista study. However, the epicatechin-enriched group did not modify the risk pattern. However, when the LS assessed CV risk, the scores of both groups corresponded to the high-risk category. While the score did not change in the placebo group, it decreased significantly in the epicatechin-enriched group.

**Figure 4:**
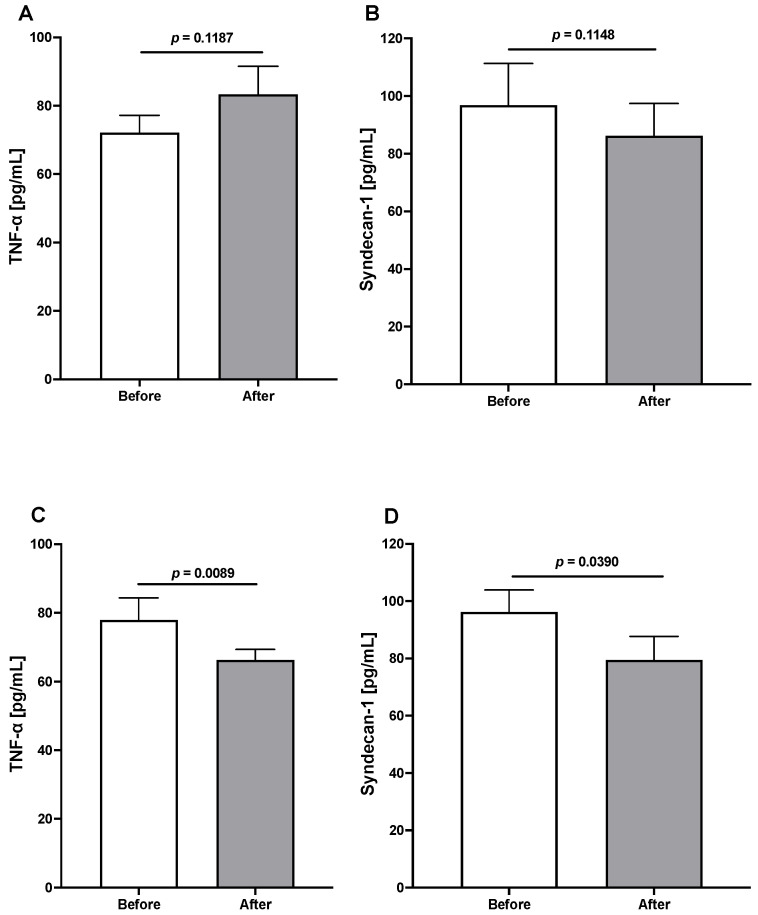
The graph shows the basal concentration of TNF-α and syndecan-1 (**A**,**B**) after three months of epicatechin-enriched treatment (**C**,**D**). In the placebo group, there was no significant change. In the actively treated group, both compounds decreased significantly. Data are expressed as the mean ± SEM, and a paired t-test was performed in each case.

On the other hand, the effect of the epicatechin-enriched regime on two markers of the glycocalyx and endothelial dysfunction, TNF-α, a proinflammatory, immunomodulator, and proapoptotic cytokine, and syndecan-1, a transmembrane protein member of the heparan sulfate proteoglycan family involved in the synthesis of heparan sulfate and other proteoglycans and a marker of endothelial glycocalyx degradation, is shown in Figure 4.

Both molecules remained without significant modifications in the control–placebo group, while in the actively treated group, the concentrations of both markers decreased significantly.

**Figure 5:**
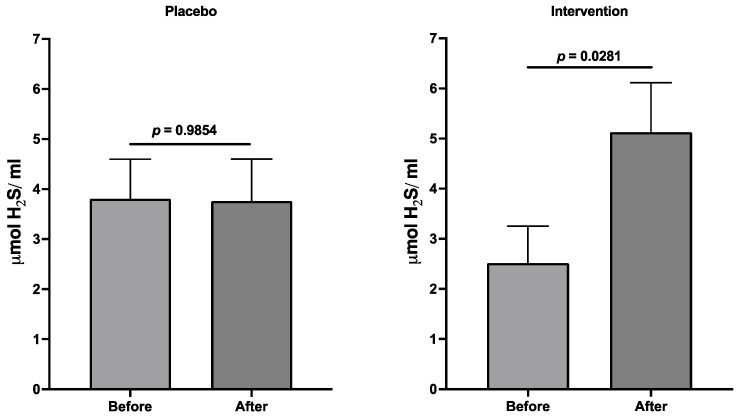
Polysulfide concentrations (as markers of an endothelial hyperpolarization relaxing factor) related to endothelial function. Data are expressed as the mean ± SEM, and a paired *t*-test was performed in each case.

Figure 5 shows the effects of both regimes on the concentration of polysulfides as markers of the gas hydrogen sulfide acting as a significant endothelium-derived hyperpolarizing factor (EDHF), whose expression parallels endothelial function. While polysulfides did not change in placebo-treated women, in those who received epicatechin-enriched treatment, the marker rose significantly, more than double.

Finally, the analysis of the oxidative damage and the change induced by treatments was explored by analyzing the concentration of malondialdehyde (MDA), a well-known marker of lipid oxidation. The results showed that the placebo induced no change in its concentration; however, the intervention with epicatechin-enriched cacao induced a decrease in MDA. Even when this result did not reach statistical significance (*p* = 0.0926), it represented a 41% decrease in the oxidative damage status (Table 3).

## 4. Discussion

The main results of this work showed that a simple, low-cost, safe **(−)**-epicatechin-enriched cacao supplement product can improve the cardiovascular risk in postmenopausal women by decreasing pro-atherogenic small and very small LDL-c particles. The results also showed that following traditional diagnosis and biochemical tests, the cardiovascular risk in these women is misjudged.

In recent decades, the art and science of cardiovascular prevention have expanded considerably to the point that in many industrialized countries and some with emerging economies, cardiovascular diseases as a whole and atherosclerotic ischemic heart disease, in particular, are in apparent decline, while they are increasing in other regions and nations, like Mexico [37,38]. Nevertheless, ischemic heart disease is still the first cause of mortality worldwide in both genders [39]. Despite all the conceptual, technological, and therapeutic achievements, there is no clear consensus regarding preventive or therapeutic recommendations in some specific populations of patients. Premenopausal, menopausal, and postmenopausal women are one of these groups that, despite the high CV risk they face, there are no categorical and specific preventive and therapeutical recommendations. The main determinant and trigger of CVD is O/O. In the climacteric and preclimacteric states, a malignant circle is established in which each of the components of the binomial aggravates the other. As the initial underlying process of abdominal O/O is the binomial insulin resistance/hyperinsulinism, MS is not only the cause of other metabolic conditions associated with atherosclerosis, such as diabetes, but it is also the primal origin of several cardiovascular syndromes, such as coronary syndromes and strokes. During perimenopause, the emergence of MS is frequent, which explains the increase in metabolic and cardiovascular risk [40]. It has been observed that the incidence of MS escalates six years before and six years after the suspension of menses [41].

Besides traditional CV risk factors shared with men, such as high low-density lipoprotein-cholesterol, smoking, type 2 diabetes mellitus, O/O, physical inactivity, unhealthy diet, abuse of alcohol consumption, and psychosocial stress, among others, women face specific factors related to their gender, to their reproductive health and pregnancy as early menarche, premature menopause, polycystic ovary syndrome, gestational diabetes, and hypertensive disorders of pregnancy. In addition, the perimenopausal period, characterized by hormonal fluctuations and other physiological changes, poses specific challenges to women’s cardiovascular health. Hormonal fluctuations, irregular menstrual cycles, and the gradual decline of estrogen levels characterize the perimenopausal phase. With the declination in estrogen activity during and after menopause, a series of unfavorable proatherogenic phenomena begin to take place, such as alterations in lipid profile, including increased LDL serum cholesterol and triglycerides, decreased HDL cholesterol, a prothrombotic milieu, and endothelial dysfunction, affecting vascular tone and increasing arterial stiffness and blood pressure, predisposing the development of atherosclerotic cardiovascular diseases (ASCVDs) [42]. CVD is responsible for one in every five female deaths, and few women recognize that heart disease is their number-one killer.

CV risk assessment, including blood pressure monitoring, correct lipid profile evaluation, and diabetes screening, is crucial. In this regard, the results reported in this pilot study suggest that the usual screening workshop for postmenopausal women could be insufficient and misleading. The standard laboratory tests done routinely could not show any flagrant metabolic abnormality or only a minor deviation from normality in many of these patients. Assessing the effect of a (−)-epicatechin-enriched cacao supplement, it did not become noticeable any modification of the concentrations of lipids, suggesting a misdiagnosis in the included postmenopausal women. However, employing a more profound lipid profile analysis with the technique of LDL particles with gas-phase electrophoresis ion mobility lipoprotein fractionation, which measures the size and number of particles, abnormalities that are not apparent with traditional screening can be detected. Soon, this approach may displace and replace the standard laboratory test in the clinical assessment of lipid profiles [27].

The main objective of this investigation was to analyze a low-cost alternative to modulated lipid alterations using a (−)-epicatechin-enriched cacao supplement. Flavonoids are polyphenolic compounds in some fruits, vegetables, wine, and cacao. The highly varied structural diversity of flavonoids and their circulating metabolites may modulate the molecular pathways involved in lipid metabolism. These substances have emerged as a potential approach to improve overall health.

Among the large family of flavonoids, flavanols have been extensively studied, mainly the stereoisomer of catechin, (−)-epicatechin, abundant in cocoa. Recent evidence suggests that this flavanol may prevent cardiometabolic disorders by stimulating endothelial function, decreasing abdominal adipose tissue, increasing insulin sensitivity, and activating AMP-activated protein kinase (AMPK) and peroxisome proliferator-activated receptor-gamma coactivator 1-alpha (PGC-1α), critical energy metabolism regulators. In this regard, we have recently shown that the intake of pure (−)-epicatechin (EC) stimulates mitochondrial biogenesis and function, improving the energetic metabolism with a decrease in triglyceride serum level [19,20,21]. Using indirect calorimetry and calculating the respiratory quotient (RQ) to determine the primary source of energy metabolism, we showed that EC lowers the RQ ratio, reflecting increased fat oxidation [19]. The positive effects of EC on the metabolism have been extensively demonstrated [20,43,44].

Recently, we explored an alternative source of this flavonoid. We use cacao pericarp flour containing epicatechin and add enough EC to have the equivalent of 25 mg of EC per serving. Our clinical experience with cacao by-products in overweight patients has demonstrated a significant decrease in TG and the TG/HDL index [21]. With these results in mind, we hypothesized that postmenopausal women with borderline metabolic syndrome would benefit from this treatment. Unfortunately, our results did not demonstrate substantial changes in TG and other lipids and lipoproteins. A possible explanation is the lack of dietary recommendations in our study protocol. However, our results show a striking reduction in highly atherogenic, very small, and small LDL particles in the (−)-epicatechin-enriched cacao-supplement-treated group. At the same time, the large and buoyant LDLs increased. These changes indicate a notorious antiatherogenic effect secondary to the EC action on improving insulin sensitivity, thus reducing the production of the smaller LDL subclasses. In addition, an important reduction in MDA, a marker of oxidative stress and a final product of lipoperoxidation, was found; these results indicate a decrease in oxidation, which is one of the first steps of atherogenesis.

Using risk scales like the one created by the American College of Cardiology/American Heart Association (ACC/AHA), with the data of populations strikingly different from ours, could signal a complete failed risk assessment. In our patients, the estimated ACC/AHA risk was very low, in contrast with the elevated figure of the TG/HDL index and the LS, the latter created with the data of contemporary middle-class Mexican mestizos, which is still under experimental proof. We acknowledged that the TG/HDL-c index and the Lindavista score has to be validated in future studies, given that there are ethnic, anthropometric, and nutritional differences among different geographic zones. This index has no universal values because it is influenced by gender, ethnicity, age, and other factors. However, we know that the higher the value of the ratio, the greater the cardiovascular and cardiometabolic risk.

In the placebo group, the CV risk value assessed with the three methods used in the study did not change throughout the trial. The ACC/AHA and the TG/HDL index did not change in the experimental group. Still, the LS was reduced significantly, probably indicating that the latter scoring system is more sensitive to detecting the risk in subjects with minor abnormalities of risk factors. The LS system’s background is that the whole CV risk is the sum of all the risk factors, the so-called “risk aggregation” [45].

In the experimental group, it was evident the reduction in the TNF-a and syndecan-1 signaling concentration had a beneficial effect on endothelial function and the structure and function of glycocalyx. In an attempt to evaluate, indirectly, endothelial function, we analyzed the levels of hydrogen sulfide (H_2_S), which is an indirect measure of the endothelial-derived hyperpolarization factor (EDHF). Our results showed a significant increase in this vasorelaxation-inducer. Altogether, these results suggest a positive effect on dyslipidemia-induced endothelial dysfunction and aligned with cardiovascular risk prevention as reported [46,47,48,49].

Our results agree with the work of Curtis P. et al., which shows that a one-year intervention with a flavan-3-ol-enriched product decreases CVD risk in postmenopausal type 2 diabetic patients (diabetes care) and improves vascular function [50].

On the other hand, a recent report (COSMOS trial) demonstrated that in a follow-up of 3.6 years, cocoa extract reduced CVD death by 27%, showing that extracts and no pure molecules can still induce cardioprotection [51].

In conclusion, the results of this proof-of-concept trial showed in a novel manner that a simple, low-cost, safe (−)-epicatechin-enriched cacao supplement product can improve the cardiovascular risk in postmenopausal women. These results open the possibility of using this product as an adjuvant in metabolic syndrome treatment.

Our results also signal the necessity of using proper methodologies to assess cardiovascular risk in women, particularly perimenopausal and postmenopausal women.

Limitations: This is a proof-of-concept study; therefore, the number of subjects is low and needs to be increased. It needs to be adjusted in the dose and length of treatment. A study where this product is combined with a statin treatment can be implemented. More work is necessary to fill the gap created by this work, and a larger clinical trial is guaranteed.

## Figures and Tables

**Table 1:** (Laboratory report). Number of lipoproteins by size and the CV risk of the study population.

Lipoprotein by Size		Reference Number of Particles		
	Study Population Particle Number Mean ± SEM	Optimal	Moderate Risk	High Risk
LDL peak size (Å)	211.20 ± 0.86	>222.90	217.40–222.90	<217.40
LDL total particles	1050 ± 36.55	<1260	1260–1538	>1538
LDL very small	239 ± 13.28	35–182		>182
LDL small	204.50 ± 10.17	<142	142–219	>219
LDL medium LDL	170.50 ± 7.35	<215	215–301	>301
LDL large	89.95 ± 5.19	>89–368		<89
HDL total particles	27,155 ± 684.10	17,063–38,995		
HDL small	27,068 ± 4874	12,602–28,643		
HDL large	4956 ± 131.60	>6729	6729–5353	<5353

Å: angstroms (1 Å = 0.1 nm), LDL: low-density lipoprotein, HDL: high-density lipoprotein. The atherogenicity of LDL depends on the number and size of its particles. The more numerous and the smaller the size of lipoproteins, the greater their atherogenicity power. Very small LDLs (subclass IV) vary from 242 to 246 Å; small (class III) from 247 to 252 Å; medium (class II) from 255 to 270 Å, and finally, large and buoyant (class I) from 260 to 275 Å [36].

**Table 2:** Anthropometric, metabolic, and blood pressure data of the study population.

Variable	Mean ± SEM	Range
Age (years)	53.30 ± 0.49	50–61
Body weight (kg)	73.80 ± 1.61	55–96
BMI (kg/m^2^)	31.00 ± 0.64	24.40–39.90
Abdominal circumference (cm)	96.30 ± 1.47	82–149
Glucose (md/dL)	102.10 ± 1.82	85–149
Cholesterol (mg/dL)	213.70 ± 5.75	125–295
LDL cholesterol (mg/dL)	126.90 ± 4.80	51.80–202.80
HDL cholesterol (mg/dL)	43.50 ± 1.37	30–72
Triglycerides (mg/dL)	214.60 ± 14.13	75–636
Systolic blood pressure (mmHg)	116.30 ± 2.70	89–158
Diastolic blood pressure (mmHg)	72.40 ± 1.20	59–97
10-year ASCVD risk	2.76 ± 0.13	2–5

BMI: body mass index, LDL: low-density lipoprotein, HDL: high-density lipoprotein, ASCVD: atherosclerotic cardiovascular disease.

**Table 3:** Anthropometric, metabolic, and blood pressure data separated by groups, before and after intervention.

Variable	Placebo Group			Intervention Group		
	Before	After	*p*	Before	After	*p*
Body weight (kg)	70.90 ± 2.08 ^#^	69.60 ± 2.12	0.020	76.48 ± 2.45 ^#^	75.43 ± 2.51	0.000
BMI (kg/m^2^)	29.61 ± 0.89	29.00 ± 0.93	0.009	32.41 ± 0.83	31.18 ± 0.85	0.020
Abdominal circumference (cm)	94.35 ± 2.25	92.70 ± 2.23	ns	98.33 ± 1.86	94.63 ± 1.78	0.005
Fasting glycemia (mg/dL)	99.10 ± 1.35	96.75 ± 1.75	ns	105.00 ± 3.20	105.10 ± 3.89	ns
Total cholesterol (mg/dL)	209.10 ± 7.00	223.40 ± 7.02	ns	218.00 ± 9.09	226.60 ± 9.14	ns
LDL-Cholesterol (mg/dL)	123.70 ± 5.42	135.30 ± 5.07	0.048	129.90 ± 7.90	132.60 ± 9.58	ns
HDL-Cholesterol (mg/dL)	46.42 ± 2.40	48.55 ± 2.70	ns	40.82 ± 1.16	41.16 ± 1.09	ns
Triglycerides (mg/dL)	193.30 ± 12.15 ^#^	204.40 ± 24.93	ns	220.8 ± 12.02 ^#^	223.1 ± 11.96	ns
Systolic blood pressure (mm Hg)	112.80 ± 3.45	113.10 ± 4.14	ns	119.60 ± 4.14	115.8 ± 2.70	ns
Diastolic blood pressure (mm Hg)	70.75 ± 1.66	70.30 ± 1.98	ns	73.90 ± 1.60	73.68 ± 1.42	ns
MDA (pmol/mg of dry weight)	24.62 ± 3.60	25.41 ± 4.09	ns	30.65 ± 6.90	18.06 ± 2.27	0.090

BMI: body mass index, LDL: low-density lipoprotein, HDL: high-density lipoprotein, MDA: malondialdehyde. # no statistically significant differences between the initial placebo group versus the initial experimental group. ns: non-statistically significant differences.

**Table 4:** Low- and high-density lipoprotein fractionation.

Percentage Change in the Number of LDL Particles				
LDL Size, (Å)	Placebo	Intervention	Δ	*p*-Value
Very small	28.80 ± 12.10	−31.66 ± 17.00	↓	0.04
Small	30.94 ± 12.19	−24.79 ± 18.80	↓	0.03
Medium	13.00 ± 9.16	27.56 ± 10.47	=	0.42
Large	−10.59 ± 3.48	36.81 ± 15.87	↑	0.03
**HDL Particles**			***p*** **-Value**	
Change in HDL particle number	−331.40 ± 1271	3066 ± 1042	0.050	
Change in large HDL particle number	−66.10 ± 165.10	441.20 ± 158.40	0.030	

LDL: low-density lipoprotein, Å: angstroms (Å/10 = I nm), HDL: high-density lipoprotein. Δ: delta is the difference between placebo and intervention group, ↑ increase, ↓ decrease.

**Table 5:** Cardiovascular risk assessment.

Variable	Placebo Group			Intervention Group		
	Before	After	*p*	Before	After	*p*
10-year ACC/AHA ASCVD risk	2.65 ± 0.16	2.85 ± 0.26	ns	2.89 ± 0.21	2.84 ± 0.21	ns
TG/HDL index	4.26 ± 0.42	5.00 ± 0.88	ns	5.68 ± 0.46	5.58 ± 0.41	ns
Lindavista risk score	10.45 ± 0.45	9.50 ± 0.80	ns	12.38 ± 0.39	9.81 ± 0.87	0.007

ACC/AHA ASCVD: American College of Cardiology/American Heart Association atherosclerotic cardiovascular disease, TG: triglyceride, HDL: high-density lipoprotein. ns: non-statistically significant differences.

## Data Availability

Data sharing not applicable.
